# Clinical Significance of and Predictive Risk Factors for the Postoperative Elevation of Carcinoembryonic Antigen in Patients With Non-Metastatic Colorectal Cancer

**DOI:** 10.3389/fonc.2021.741309

**Published:** 2021-10-07

**Authors:** Siyu Zhou, Nengquan Sheng, Jiazi Ren, Qian He, Yaya Zhang, Jianfeng Gong, Zhigang Wang

**Affiliations:** ^1^ Department of Gastrointestinal Surgery, Shanghai Jiao Tong University Affiliated Sixth People’s Hospital, Shanghai, China; ^2^ College of Clinical Medicine, Shanghai Jiao Tong University School of Medicine, Shanghai, China

**Keywords:** colorectal cancer, postoperative carcinoembryonic antigen, prognosis, surveillance, nomogram

## Abstract

**Background:**

Recently, a few researches focus on the correlation between postoperative carcinoembryonic antigen (post-CEA) and the outcome of colorectal cancer (CRC), but none investigates the predictive value of post-CEA in a prognostic model. Besides, current recommendations on the frequency of post-CEA surveillance are not individualized and well followed. There is an absence of identification of patients who are more likely to have abnormal post-CEA levels and need more frequent CEA measurements.

**Methods:**

Consecutive CRC patients who underwent curative surgery were enrolled and randomly divided into the discovery (n=352) and testing cohort (n=233). Impacts of preoperative CEA (pre-CEA) and post-CEA on prognosis were assessed. Cox regression model was applied to develop prognostic nomograms, which were validated by the concordance index (C-index), calibration curve, and receiver operating characteristic curve (ROC) analysis. And prediction improvement of the nomograms was assessed with net reclassification improvement (NRI) and integrated discrimination improvement (IDI). Logistic regression was used to identify predictive risk factors and construct the prediction model for post-CEA elevation.

**Results:**

Post-CEA independently predicted overall survival (OS) and disease-free survival (DFS), while pre-CEA did not. Post-CEA elevation represented higher risks in patients with normal pre-CEA, compared to those with persistent elevated CEA. The nomograms for OS and DFS were established with body mass index, tumor differentiation, N stage, lymphocyte-to-monocyte ratio, and post-CEA. The nomograms showed good calibration and superior discrimination than pTNM stage, with the C-index of 0.783 and 0.759 in the discovery set and 0.712 and 0.774 in the testing set for OS and DFS, respectively. Comparisons between models using IDI and NRI implied that the nomograms performed better than pTNM stage and the predictive power could be improved with the addition of post-CEA. The prediction model for post-CEA elevation was established with age, platelet-to-lymphocyte ratio, preoperative CA19-9, and pre-CEA. The AUC of the model in the two cohorts was 0.802 and 0.764, respectively.

**Conclusions:**

Elevated post-CEA was a strong indicator of poor prognosis. The addition of post-CEA significantly enhanced the performance of prognostic nomograms. And the prediction model for post-CEA elevation may help identify patients who ought to reasonably receive more intensive postoperative surveillance of CEA levels.

## Introduction

Colorectal cancer (CRC) is one of the most frequently diagnosed and the deadliest malignant carcinomas globally (1). Despite improvement in surgical procedures and novel treatments, the outcomes of CRC patients remain unsatisfactory. Approximately 15% of patients in stage II and 30% of patients in stage III will experience relapse after curative resection, with most recurrence arising in the liver and lungs (2, 3). Prognosis classification of CRC is traditionally based on the pathological stage of the tumor (4), but its accuracy has been called into question recently since tumors with identical stages can display distinct clinical behaviors and outcomes (5, 6). Thus, more biomarkers are needed to complement the TNM staging system.

Carcinoembryonic antigen (CEA) is a glycoprotein associated with carcinogenesis (7). Several guidelines recommend CEA as a biomarker for CRC prognosis and suggest that CEA should be routinely measured in patients with non-metastatic CRC (8, 9). It has been known that elevated preoperative CEA (pre-CEA) is related to distant metastasis or worse outcomes of CRC (10, 11), but some researchers propose that the sensitivity and specificity of pre-CEA in identifying high-risk CRC patients are limited (12, 13). Recently, a few studies focus on the prognostic role of the postoperative CEA (post-CEA) level and illustrated that post-CEA was a more valuable biomarker than pre-CEA in distinguishing CRC prognosis (14–16). In addition, although prognostic models and nomograms are recommended for predicting recurrence and survival in various malignancies due to its practicability and comprehensiveness (17), no published research has considered and incorporated post-CEA in the prognostic model for stages I–III CRC. Furthermore, in spite of the recommendation by the practice guideline that post-CEA examination should be performed every 6 months for all patients who undergo curative surgery of CRC (3), this suggestion on surveillance is not individualized.

Therefore, the main purpose of this study is to establish a robust nomogram to predict the outcome of CRC, with the utilization of the prognostic value of post-CEA level. Besides, we aim to investigate the risk factors for post-CEA elevation and identify patients who should receive earlier and more intensive measurements of postoperative serum tumor markers.

## Materials and Methods

### Patient Cohort

We retrospectively reviewed consecutive patients diagnosed with CRC who received curative operations between January 2015 and December 2018 in the General Surgery Department, Shanghai Jiao Tong University Affiliated Sixth People’s Hospital. The inclusion criteria were listed below: (a) histologically confirmed primary malignancies of the colon and rectum; (b) radical resection (R0) of the tumor was performed. And patients who met the following criteria were excluded: (a) with metastasis; (b) with preoperative neoadjuvant chemotherapy; (c) without CEA measurement before or after surgery; (d) follow-up time was less than 3 months. A total of 585 patients were enrolled and randomly divided into the discovery and testing cohort using a split proportion of 6:4. This study was approved by the ethics committee of the hospital, and the patient’s consent was obtained.

Demographic and clinical data of the patients were retrieved from the medical records, including gender, age, body mass index (BMI), bowel obstruction, operation type, TNM stage, the number of harvested lymph nodes (LNs), tumor site, tumor size, histological type and degree of differentiation of the tumor, lymphovascular invasion, perineural invasion, and the status of KRAS mutation and mismatch repair (MMR). BMI was classified according to the WHO criteria (18), and tumors were graded in accordance with the guidance of AJCC version 8 (19). Preoperative serum tumor markers and other laboratory indices were measured within 7 days before surgery. Post-CEA was defined as the last measurement within 12 weeks after operation and before chemotherapy. Systemic inflammatory indicators including neutrophil-to-lymphocyte ratio (NLR), platelet-to-lymphocyte ratio (PLR), and lymphocyte-to-monocyte ratio (LMR) were calculated in accordance with previous literature (20). The cutoff value of the inflammation-related index was obtained by the receiver operator characteristic curve (ROC). And the upper reference limit for CEA (preoperative and postoperative), CA125, and CA19-9 were 5 ng/ml, 35 U/ml, and 27 U/ml, respectively, according to which tumor markers were categorized into a normal or elevated level.

All patients were followed up regularly *via* telephone interviews, and the status of survival and relapse was recorded. Overall survival (OS) was defined as the time from surgery to death of any cause. Disease-free survival (DFS) was defined as the time from surgery to recurrence.

### Statistical Analysis

All analyses were conducted with R software, version 4.0.3. Differences between groups were compared with the Pearson chi-square test or *Fisher* exact test. Kaplan-Meier method was applied to calculate the rate of OS and DFS, and a log-rank test was used to assess the difference between groups. The effect of pre-CEA and post-CEA on OS and DFS in distinct subgroups of CRC patients was investigated and summarized in the form of a forest plot. The candidates of prognostic predictors were analyzed in the univariate and multivariate Cox proportional hazard regression in the discovery cohort, to establish the prognostic models for predicting the OS rate and DFS rate at 1 year, 3 years, and 5 years. Furthermore, univariate and multivariate logistic regression methods were used in the discovery set to explore the risk factors for and construct the prediction model for the post-CEA elevation. The prognostic models were validated by the Harrell’s concordance index (C-index), calibration curve, and ROC curve analysis. Performance improvement of the nomogram compared with pTNM stage and non-CEA nomogram was evaluated by computing the net reclassification improvement (NRI) and integrated discrimination improvement (IDI) on 1,000 bootstrap samples. The prediction model for post-CEA elevation was evaluated *via* ROC curve, calibration curve, and the Hosmer-Lemeshow goodness-of-fit test. A two-tailed P < 0.05 was deemed statistically significant in all tests.

## Results

### Patient Characteristics

A total of 585 subjects were included in this research, which comprised 340 (58.1%) men and 245 (41.9%) women with a median age of 65 years (range, 23–92 years). According to the AJCC staging system, there were 88 (15.0%), 228 (39.0%), and 269 (46.0%) patients classified as clinical stage I, stage II, and stage III, respectively. Tumors were located in the colon in 374 (63.9%) patients and the rectum in 211 (36.1%) patients. Elevation of pre-CEA and post-CEA occurred in 221 (36.1%) and 89 (15.2%) patients. The median follow-up period was 38 months (range 4–72 months). At the end of the follow-up, 104 (17.8%) patients died and 128 (21.9%) patients underwent recurrence. The 3-year OS rate in all patients was 85.5% (95% CI: 82.6–86.6%), and the 3-year DFS rate was 0.798 (95% CI: 0.765–0.832). These patients were randomly separated into a 60% discovery (n=352) cohort and a 40% testing (n=233) cohort. No significant difference was found between the two cohorts, as described in **Supplementary Table S1**.

The correlation between post-CEA levels and the clinical features of all enrolled patients was presented in **Supplementary Table S2**. It was demonstrated that post-CEA elevation was more frequent in patients with advanced age (P<0.001), right-side colon cancer (P=0.024), positive lymphovascular invasion (P=0.045), high grade of T (P=0.026) and N (P=0.023) stage, elevated NLR (P=0.016) and PLR (P<0.001), and positive preoperative CEA (P<0.001) and CA19-9 (P<0.001). The post-CEA declined to a normal level after the curative surgery in 156 (70.6%) of the 221 patients with elevated pre-CEA. Besides, among the 364 patients with normal pre-CEA, a small proportion (6.6%) even presented increment in post-CEA. Death was observed in 38 (43%) and 66 (13%) cases with an elevated and normal level of post-CEA, respectively. To note, relapse occurred in 42 (47%) cases with elevated post-CEA, of whom over a half (22 out of 42, 52.4%) had recurrence within 1 year after operation, while only 86 (17%) patients with normal post-CEA suffered from recurrence, and 40 (46.5%) of the 86 patients had recurrence within 1 year.

### Prognostic Significance of Pre-CEA and Post-CEA in CRC Patients

**Figure 1:**
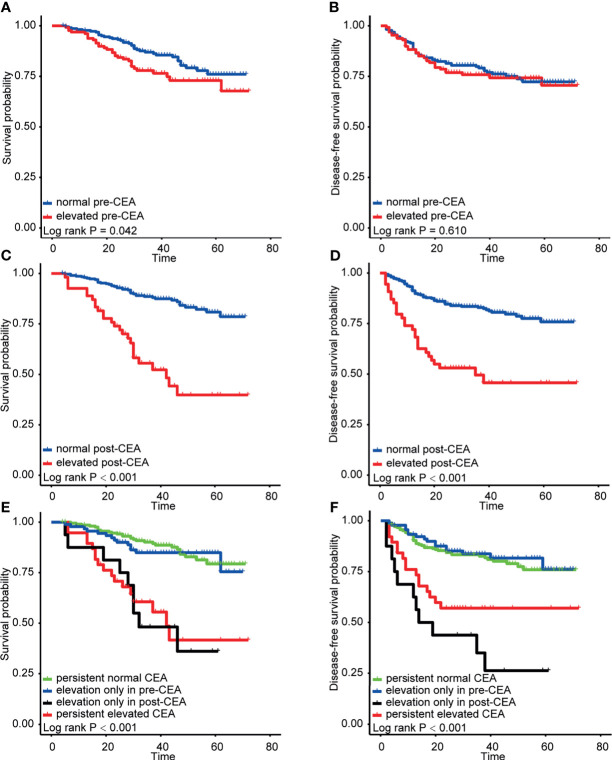
Kaplan-Meier analysis for patients with colorectal cancer (CRC) in the discovery cohort stratified by preoperative carcinoembryonic antigen (pre-CEA) and postoperative carcinoembryonic antigen (post-CEA). **(A, C, E)** were the survival curves for overall survival (OS). **(B, D, F)** were the survival curves for disease-free survival (DFS).

The Kaplan-Meier method and log-rank test were applied in the discovery cohort to assess the effect of pre-CEA and post-CEA on survival and recurrence (**Figure 1**). Our results suggested that pre-CEA was capable of distinguishing patients in OS (P=0.042), but not in DFS (P=0.610). Besides, when compared to patients who had normal post-CEA, those with elevated post-CEA presented significant worse OS (P<0.001) and DFS (P<0.001), with a lower 3-year OS rate [0.556 (0.430–0.718) *vs.* 0.885 (0.849–0.924)] and 3-year DFS rate [0.495 (0.372–0.660) *vs.* 0.829 (0.787–0.874)].

When analyzing pre-CEA and post-CEA in combination, we found the CEA level that increased preoperatively but normalized postoperatively exerted a similar impact on OS (P=0.560) compared with consistently normal CEA level. There was also no significant difference in OS between patients whose CEA remained elevated all along and patients with negative pre-CEA but elevated post-CEA (P=0.850). Furthermore, among the subset of patients with normal post-CEA, those of different pre-CEA levels had indistinguishable DFS (P=0.720). And our results interestingly revealed that it was the patient with negative pre-CEA that turned into positive after surgery who trended to have the worst DFS.

### Effect of Post-CEA on Prognosis in Subgroups

**Figure 2:**
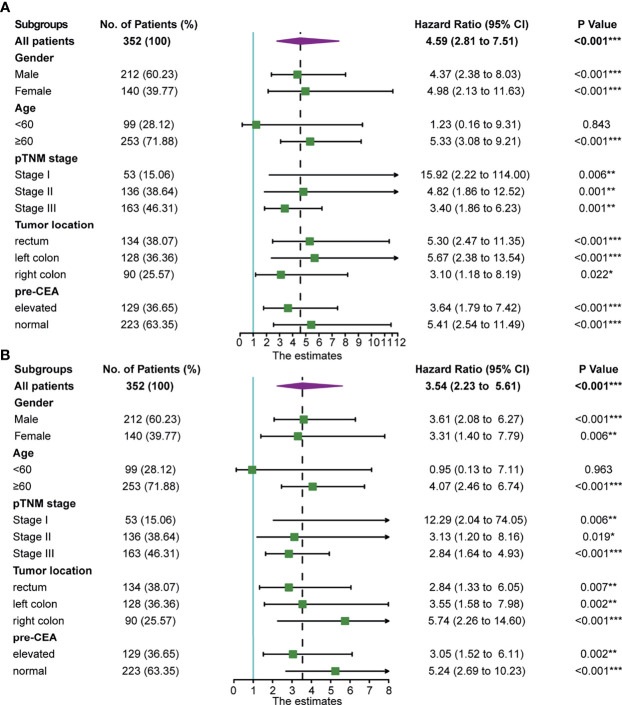
Association of postoperative carcinoembryonic antigen (post-CEA) with **(A)** overall survival (OS) and **(B)** disease-free survival (DFS) in all patients and subgroups of patients with colorectal cancer (CRC) in the discovery cohort. Pre-CEA, preoperative carcinoembryonic antigen; CI, confidence interval. (*P < 0.05, **P < 0.01, ***P < 0.001).

The subgroup analysis of the prognostic value of post-CEA in the discovery cohort was performed and shown in **Figure 2**. As illustrated by the forest plot, post-CEA was an indicator for poor OS in most of the subgroups, including different genders, TNM stages, tumor locations, and pre-CEA levels. Notably, elevation in post-CEA represented a close risk for death in men and women, and a higher hazard ratio (HR) for OS in patients with normal pre-CEA compared with patients with persistently elevated CEA [HR: 5.41 (2.54–11.49) *vs.* 3.64 (1.79–7.42)]. We got semblable results for DFS. Post-CEA elevation was associated with poor DFS in most subgroups with the exception of younger patients (age <60). The HRs of post-CEA for DFS were also different between the two pre-CEA levels [normal pre-CEA: 5.24 (2.69–10.23); elevated pre-CEA: 3.05 (1.52–6.11)].

### Univariate and Multivariate Analyses of Factors Associated With OS and DFS

In order to investigate the independent prognostic factors for OS and DFS, univariate and multivariate Cox proportional hazard regression were used in the discovery cohort. It was found that BMI [normal, HR: 0.308 (0.148–0.642), P=0.002; overweight, HR: 0.368 (0.157–0.862), P=0.021], degree of tumor differentiation [HR: 1.774 (1.056–2.980), P=0.030], N stage [N1, HR: 2.872 (1.413–5.841), P=0.004; N2, HR: 4.633 (1.873–11.457), P<0.001), LMR [HR: 0.358 (0.175–0.734), P=0.005], and post-CEA level [HR: 3.614 (2.045–6.388), P<0.001] were significant independent variables associated with OS (**Supplementary Table S3**). Similarly, BMI [normal, HR: 0.448 (0.207–0.969, P=0.041; overweight, HR: 0.393 (0.162–0.956), P=0.039], degree of tumor differentiation [HR: 1.667 (1.051–2.643), P=0.030], N stage [N1, HR: 2.792 (1.458–5.349), P=0.002; N2, HR: 6.133 (2.781–13.526), P<0.001], LMR [HR: 0.392 (0.205–0.748), P=0.005], and post-CEA level [HR: 3.072 (1.861–5.073), P<0.001] were significant independent variables associated with DFS (**Supplementary Table S4**). Although pre-CEA was significantly associated with DFS in the univariate analysis, it was not an independent predictor after adjusting for other variables.

### Development and Validation of the Prognostic Nomograms

**Figure 3:**
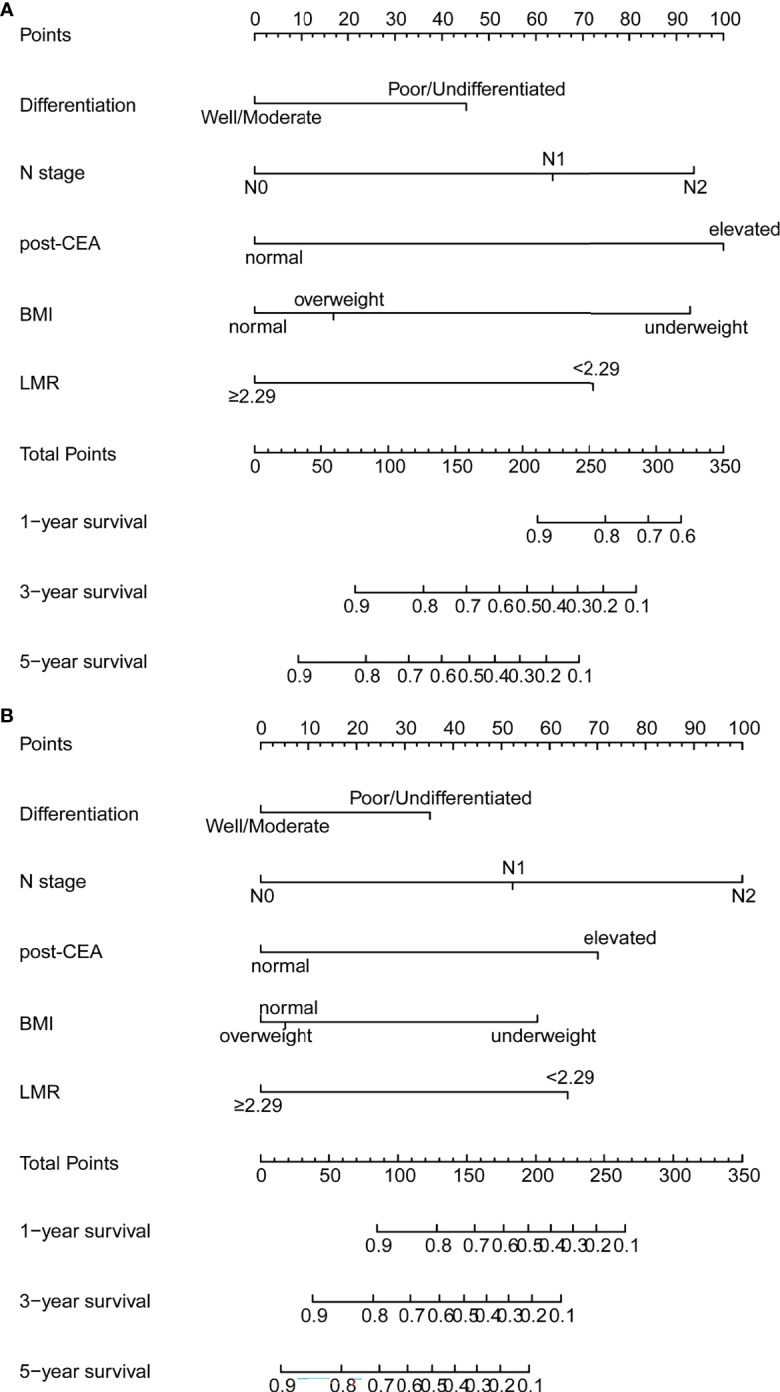
Prognostic nomograms for prediction of the 1-year, 3-year, and 5-year **(A)** overall survival (OS) and **(B)** disease-free survival (DFS) of patients with colorectal cancer (CRC). post-CEA, post-operative carcinoembryonic antigen; BMI, body mass index; LMR, lymphocyte-to-monocyte ratio.

According to the multivariate analysis, the nomograms for predicting OS and DFS were constructed (**Figure 3**). The two models incorporated five identical variables, including BMI, tumor differentiation, N stage, LMR, and post-CEA level, but the coefficients assigned to each variable were distinct between the two models and were based on the hazard ratios in the multivariate Cox regression. The predicted OS and DFS rates of an individual patient were calculated from the nomogram as follows. Each prognostic feature was translated into a corresponding risk score based on the actual value of the variable, and the total points were the sum of all items. According to the bottom scale, the 1-, 3-, and 5-year OS and DFS predictions could be obtained by the total points. For example, a patient with poorly differentiated tumor (score 45.2 for OS; 35.2 for DFS), N0 (score 0 for OS; 0 for DFS), normal BMI (score 0 for OS; 5.1 for DFS), high level of LMR (score 0 for OS; 0 for DFS), and elevated post-CEA (score 100 for OS, 70.1 for DFS) would have a total risk point of 145.2 for OS and 110.4 for DFS, indicating the 1-year, 3-year, and 5-year OS rates of 0.96, 0.75, and 0.58, and 1-year, 3-year, and 5-year DFS rates of 0.85, 0.69, and 0.58, respectively. In addition, to facilitate the application of the nomograms, two web-based dynamic calculators of survival rates were built up. Users were able to easily acquire the estimated survival rates by simply inputting the actual levels of the variables. The dynamic calculators could be accessed at https://medicaltools.shinyapps.io/Predict_OS_in_CRC/ and https://medicaltools.shinyapps.io/Predict_DFS_in_CRC/.

The model-fitting performance of the models was evaluated by C-index. The C-indices of pre-CEA, post-CEA, nomogram, non-CEA nomogram, and pTNM stage are summarized in **Table 1**. In regard to predicting OS, the predictive power of post-CEA was slightly greater than pre-CEA, although not statistically significant (P>0.05). Besides, the nomogram had the highest C-index both in the discovery (C-index: 0.783, 95% CI: 0.757–0.809) and testing (C-index: 0.712, 95% CI: 0.667–0.757) cohort, followed by the non-CEA nomogram and pTNM stage. Similar results were obtained for DFS. The post-CEA had a significantly higher C-index than pre-CEA in the discovery group, but not in the testing group. The C-index of the nomogram for DFS was 0.759 (95% CI: 0.733–0.785) and 0.774 (95% CI: 0.741–0.807) in the two cohorts, respectively, and was more preferable than pTNM stage (P<0.001). Furthermore, in the discovery cohort the predictive ability was significantly improved by the addition of post-CEA to the nomogram (P=0.037).

**Table 1:** C-indexes of prognostic factors or models for predicting OS and DFS in the discovery and testing cohort.

Factors or models	Discovery cohort		Testing cohort	
	C-index (95% CI)	P value	C-index (95% CI)	P value
OS				
Pre-CEA	0.574 (0.542–0.606)		0.565 (0.520–0.610)	
Post-CEA	0.640 (0.610–0.670)		0.633 (0.590–0.676)	
Nomogram	0.783 (0.757–0.809)		0.712 (0.667–0.757)	
Non-CEA nomogram	0.721 (0.690–0.752)		0.657 (0.609–0.705)	
pTNM stage	0.652 (0.625–0.679)		0.622 (0.581–0.663)	
Pre-CEA *vs.* Post-CEA		0.077		0.190
Nomogram *vs.* non-CEA nomogram		0.008		0.046
Nomogram *vs.* TNM stage		<0.001		0.019
DFS				
Pre-CEA	0.517 (0.490–0.544)		0.537 (0.498–0.576)	
Post-CEA	0.612 (0.587–0.637)		0.603 (0.568–0.638)	
Nomogram	0.759 (0.733–0.785)		0.774 (0.741–0.807)	
Non-CEA nomogram	0.726 (0.699–0.753)		0.732 (0.694–0.770)	
pTNM stage	0.645 (0.619–0.671)		0.668 (0.634–0.702)	
Pre-CEA *vs.* Post-CEA		0.002		0.135
Nomogram *vs.* non-CEA nomogram		0.036		0.052
Nomogram *vs.* TNM stage		<0.001		<0.001

C-index, concordance index; OS, overall survival; DFS, disease-free survival; CI, confidence interval; pre-CEA, preoperative carcinoembryonic antigen; post-CEA, post-operative carcinoembryonic antigen; BMI, body mass index; LMR, lymphocyte-to-monocyte ratio.

Nomogram: BMI + Differentiation + LMR + N stage + post-CEA.

Non-CEA nomogram: BMI + Differentiation + LMR + N stage.

Additionally, the results of NRI and IDI suggested that our nomogram had superior accuracy than pTNM stage (NRI>0, IDI>0) in OS and DFS estimation (**Table 2**). And there was also a trend that the nomogram performed better than the non-CEA nomogram, but the improvement provided by post-CEA did not always reach statistical significance for predicting CRC outcomes at different survival times (**Table 3**).

**Table 2:** Improvement of the nomogram for predicting OS compared with pTNM stage and non-CEA nomogram.

	Survival Time	Items	Discovery cohort		Testing cohort	
			Estimate (95% CI)	P value	Estimate (95% CI)	P value
Nomogram *vs.* pTNM stage	1-year	IDI	0.054 (0.009–0.190)	0.004	0.074 (0.009–0.421)	0.016
		NRI	0.311 (–0.030–0.683)	0.070	0.378 (0.014–0.690)	0.046
	3-year	IDI	0.169 (0.103–0.289)	<0.001	0.131 (0.057–0.349)	<0.001
		NRI	0.381 (0.224–0.589)	0.002	0.319 (0.172–0.592)	<0.001
	5-year	IDI	0.199 (0.114–0.310)	0.002	0.093 (0.015–0.259)	0.018
		NRI	0.479 (0.144–0.605)	0.010	0.189 (−0.142–0.459)	0.336
Nomogram *vs.* non-CEA nomogram	1-year	IDI	0.039 (−0.003–0.127)	0.080	0.040 (−0.012–0.194)	0.202
		NRI	0.297 (0.007–0.633)	0.042	0.287 (−0.142–0.626)	0.158
	3-year	IDI	0.080 (0.022–0.162)	0.004	0.060 (0.001–0.185)	0.036
		NRI	0.306 (0.180–0.445)	0.004	0.288 (−0.065–0.480)	0.076
	5-year	IDI	0.078 (0.017–0.156)	0.004	0.063 (0.000–0.172)	0.048
		NRI	0.242 (0.085–0.414)	0.018	0.196 (−0.075–0.414)	0.112

IDI, integrated discrimination improvement index; NRI, category-less net reclassification index; OS, overall survival; CI, confidence interval.

Nomogram: BMI + Differentiation + LMR + N stage + post-CEA.

Non-CEA nomogram: BMI + Differentiation + LMR + N stage.

**Table 3:** Improvement of the nomogram in predicting DFS compared with pTNM stage and non-CEA nomogram.

	SurvivalTime	Items	Discovery cohort		Testing cohort	
			Estimate (95% CI)	P value	Estimate (95% CI)	P value
Nomogram *vs.* pTNM stage	1-year	IDI	0.123 (0.056–0.256)	<0.001	0.098 (0.039–0.244)	<0.001
		NRI	0.382 (0.179–0.563)	<0.001	0.346 (0.138–0.637)	0.008
	3-year	IDI	0.171 (0.107–0.274)	<0.001	0.145 (0.083–0.282)	<0.001
		NRI	0.466 (0.232–0.570)	<0.001	0.340 (0.168–0.607)	<0.001
	5-year	IDI	0.134 (0.062–0.237)	0.002	0.137 (0.066–0.269)	0.004
		NRI	0.387 (0.035–0.543)	0.038	0.168 (−0.047–0.631)	0.096
Nomogram *vs.* non-CEA nomogram	1-year	IDI	0.067 (0.015–0.147)	0.008	0.020 (−0.023–0.112)	0.378
		NRI	0.286 (0.095–0.464)	0.012	0.227 (−0.112–0.459)	0.118
	3-year	IDI	0.067 (0.023–0.132)	0.002	0.035 (−0.007–0.121)	0.130
		NRI	0.271 (0.146–0.396)	0.004	0.217 (−0.012–0.390)	0.060
	5-year	IDI	0.033 (−0.01–0.093)	0.126	0.072 (0.006–0.169)	0.028
		NRI	0.159 (−0.06–0.310)	0.100	0.327 (0.022–0.540)	0.044

IDI, integrated discrimination improvement index; NRI, category-less net reclassification index; DFS, disease-free survival; CI, confidence interval.

Nomogram: BMI + Differentiation + LMR + N stage + post-CEA.

Non-CEA nomogram: BMI + Differentiation + LMR + N stage.

**Figure 4:**
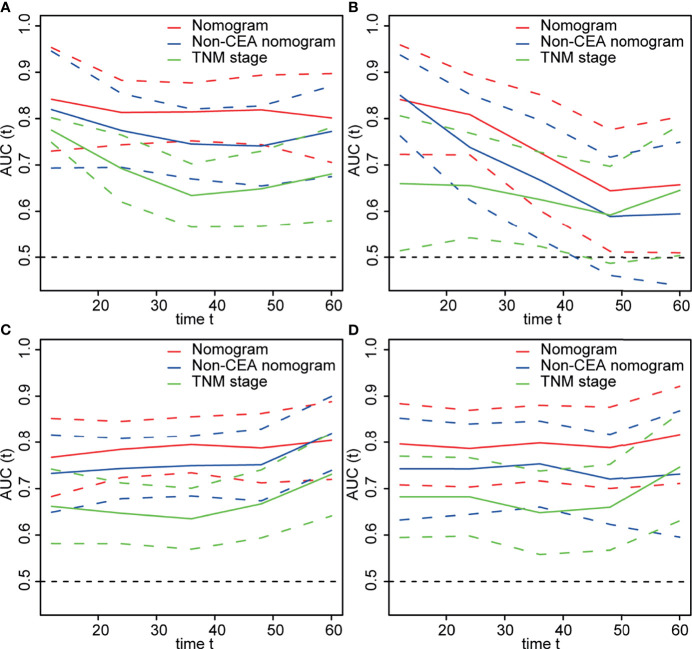
The area under time-dependent receiver operator characteristic curve (ROC) of models for predicting survival in the **(A)** discovery and **(B)** testing cohorts and predicting recurrence in the **(C)** discovery and **(D)** testing cohorts. Nomogram: Body mass index (BMI) + differentiation + N stage + lymphocyte-to-monocyte ratio (LMR) + postoperative carcinoembryonic antigen (post-CEA). Non-CEA nomogram: BMI + differentiation + N stage + LMR.

**Figure 5:**
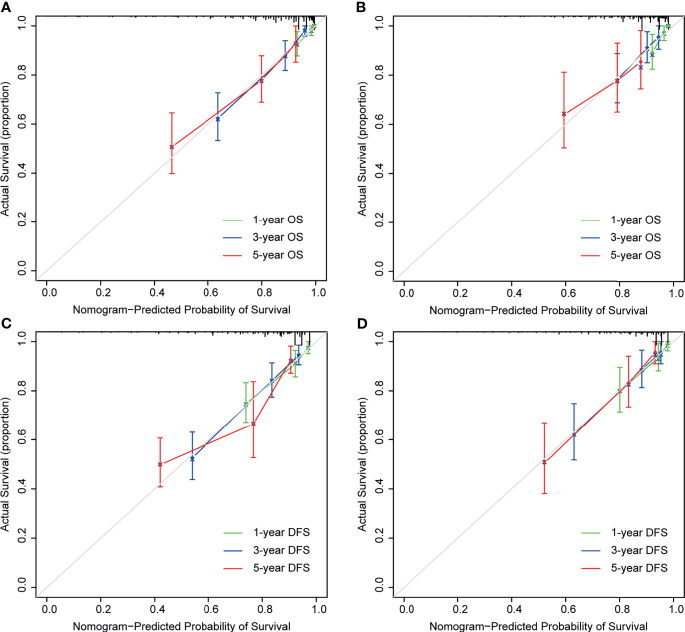
Calibration curves for nomogram predictions. The calibration curves for predicting 1-, 3-, and 5-year overall survival (OS) in the discovery **(A)** and testing cohort **(B)**. The calibration curves for predicting 1-, 3-, and 5-year disease-free survival (DFS) in the discovery **(C)** and testing cohort **(D)**.

As shown in **Figure 4**, the time-dependent ROC curves implied that within a wide range of periods, the discriminative ability of the nomograms for survival and recurrence was superior to the non-CEA nomogram as well as the AJCC TNM staging system. In the discovery set, the area under the curve (AUC) of the nomogram was 0.814 (95% CI: 0.752–0.877) for 3-year OS and 0.795 (95% CI: 0.734–0.856) for 3-year DFS. In the testing set, the AUC of the nomogram was 0.727 (95% CI: 0.602–0.852) for 3-year OS and 0.798 (95% CI: 0.716–0.880) for 3-year DFS. In addition, the calibration plot of the nomogram showed favorable agreement between the prediction and actual observation of 1-year, 3-year, or 5-year OS and DFS (**Figure 5**).

### Risk Factors and Prediction Model for the Postoperative Elevation of CEA

**Figure 6:**
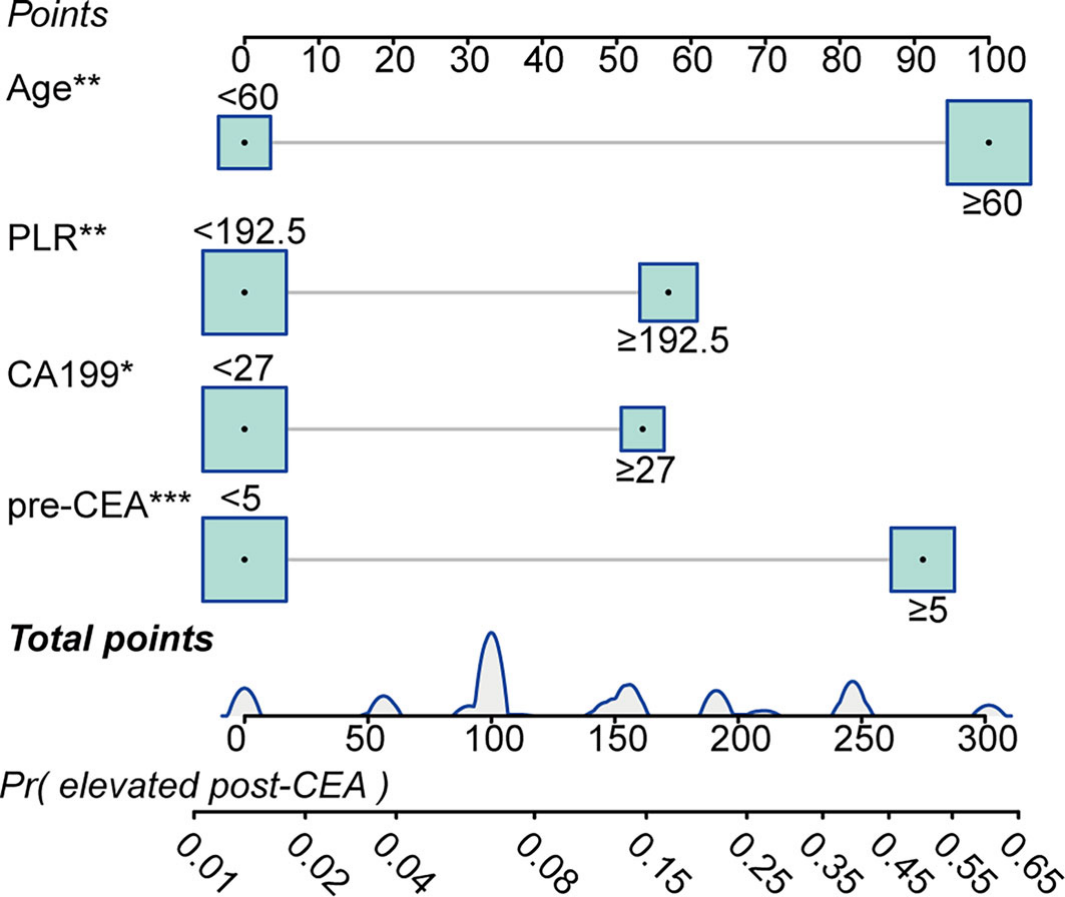
Predictive model for predicting postoperative carcinoembryonic antigen (post-CEA) elevation in patients with colorectal cancer (CRC). PLR, platelet-to-lymphocyte ratio; pre-CEA, preoperative carcinoembryonic antigen; Pr, probability of.

We performed univariate and multivariate logistic regression methods to screen out factors which predicted post-CEA elevation. As shown in **Supplementary Table S5**, it was found advanced age [OR: 4.67 (1.844–14.531), P=0.003], elevated pre-CEA [OR: 4.097 (2.082–8.328), P<0.001], elevated preoperative CA19-9 [OR: 2.294 (1.138–4.583), P=0.019, and a high level of PLR [OR: 2.168 (1.016–4.657), P=0.045] were independent predictors for elevated post-CEA. Accordingly, these variables were used to develop the prediction model for post-CEA elevation. As presented in **Figure 6**, the total points of an individual patient were calculated as [advanced age (age>60)]*100+(elevated pre-CEA)*91.1+(elevated preoperative CA19-9)*53.5+(high level of PLR)*57.0. And based on the total points, the probability of post-CEA elevation could be obtained on the risk scale bar.

**Figure 7:**
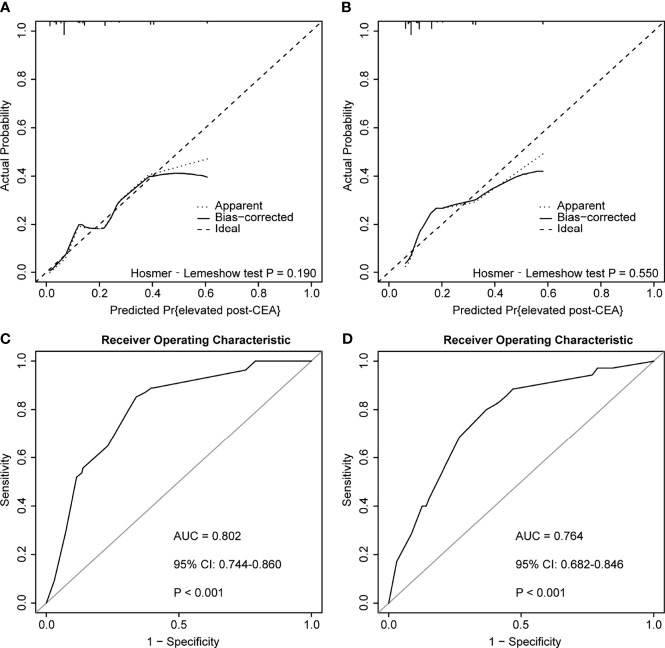
Calibration curves and receiver operator characteristic curves (ROC) of the predictive model for predicting postoperative carcinoembryonic antigen (post-CEA) elevation in patients with colorectal cancer (CRC). **(A, B)** represented calibration curves of the model in the discovery and testing cohort, respectively. **(C, D)** represented ROC curves of the model in the discovery and testing cohort, respectively. AUC, area under the curve; CI, confidence interval.

According to the ROC plot (**Figure 7**), the model showed satisfying predictive accuracy with the AUC of 0.802 (95% CI: 0.744–0.860) in the discovery cohort and 0.764 (95% CI: 0.682–0.846) in the testing cohort. The prediction model also presented adequate calibration, according to the calibration curve and the Hosmer-Lemeshow goodness-of-fit test, but the false-positive rate may increase when the actual probability of post-CEA elevation was over 0.4.

## Discussion

Although recent researches illustrated the correlation between post-CEA and CRC prognosis, none of them used post-CEA to develop CRC prediction model, and we expected that the addition of post-CEA level would increase the prediction accuracy of CRC outcome. Besides, despite the great significance of postoperative surveillance of CEA, there was an absence of personalized advice on the measurement frequency. In our study, we assessed and compared the prognostic value of pre-CEA and post-CEA in non-metastatic CRC, and established the predictive nomograms for OS and DFS with independent prognostic variables including BMI, N stage, tumor differentiation, LMR, and post-CEA. The nomograms showed good calibration and superior discrimination and prediction accuracy than TNM staging system, according to the C-index, NRI, IDI, and the AUC value (**Tables 1**–**3** and **Figure 4**). By comparing nomogram and non-CEA nomogram, our speculation was confirmed that post-CEA level was important in the construction of the model and significantly improved the performance of the nomogram. Furthermore, our results also demonstrated that age, PLR, preoperative CA19-9, and pre-CEA were independently associated with post-CEA elevation, and a prediction model developed with these factors had an AUC of 0.802 (95% CI: 0.744–0.860) in the discovery cohort and 0.764 (95% CI: 0.682–0.846) in the testing cohort. This was the first study that incorporated post-CEA in prognostic models for predicting OS and DFS in patients with non-metastatic CRC. Additionally, this study also looked into the risk factors for post-CEA elevation and identified the patients who may benefit from more intensive follow-up of serum tumor markers for the first time.

For patients with stages I–III CRC, elevated pre-CEA could normalize after the curative surgery (21–23). But in clinical practice, CEA normalization was not observed in approximately 30% of the patients (15), and a high level of post-CEA implied the existence of minimal residual disease (MRD), which increased the risk of relapse after operation (24, 25). In addition to this, circulating tumor DNA (ct-DNA), which derived from tumor cells, was also reported to correlate with the presence of MRD and have a better predictive power for CRC prognosis than post-CEA. But its application was limited as a result of the high cost (26). In contrast, CEA detection was cheaper and easier to implement. Thus, pre-CEA was widely used in distinguishing the outcomes of patients with CRC, while recent studies underlined the great significance of postoperative surveillance of CEA. You et al. found that elevated post-CEA and post-CEA increment were independently associated with OS and PFS in stages II–III CRC patients (27). Additionally, the ratio of post-CEA to pre-CEA less than 0.5 was reported as an indicator of prolonged survival in CRC, especially in patients with positive pre-CEA (28). Konishi et al. discovered that patients with post-CEA elevation had a greater probability for earlier relapse and were more vulnerable in the first 12 months after curative surgery. They also found that post-CEA was more informative than pre-CEA, and pre-CEA was not prognostic in patients with elevated post-CEA (15). Consistent with these studies, our findings revealed that post-CEA was an independent prognostic factor for both OS and DFS while pre-CEA was not, according to the multivariate analysis. Similar outcomes were observed between patients who had consistent normal CEA and patients with normalized post-CEA, indicating that post-CEA was more prognostic and important than pre-CEA, which was also confirmed by the comparison using C-indices.

Moreover, our results of subgroup analysis demonstrated the correlation between post-CEA and CRC prognosis was independent of sexes, tumor stages, tumor locations, and pre-CEA levels. The correlation was not significant in younger patients (age <60), and one of the possible reasons for this was that the ratio of elevated post-CEA was extremely low (5 out of 99, 5.05%) among CRC patients aged less than 60 years old. To note, we interestingly found that post-CEA elevation represented greater risk in the subgroup with normal pre-CEA than those with elevated pre-CEA both for OS [HR: 5.41 (2.54–11.49) *vs.* 3.64 (1.79–7.42)] and DFS [HR: 5.24 (2.69–10.23) *vs.* 3.05 (1.52–6.11)]. It was the first report that the subset of patients with normal pre-CEA turning elevated after surgery was most vulnerable to more rapid death and relapse. In addition, although Sonoda et al. concluded early-stage CRC patients did not need post-CEA measurements due to the low risk of disease (29), in the present study, elevated post-CEA was associated with worse OS and DFS in stage I CRC. Therefore, we believed post-CEA should be detected in all patients with non-metastatic CRC, irrespective of the tumor stage, and should be considered when determining treatment intensity and duration.

Nowadays, most of the guidelines recommended a postoperative detection of CEA every 3–6 months in patients who underwent radical resection of CRC (3, 9). However, current recommendations on surveillance were not well followed by patients, and more than one-quarter of them did not receive CEA measurements within 12 to 18 months after operation (28, 30, 31). Although a meta-analysis of randomized trials implied that more intensive follow-up contributed to prolonged survival of patients with CRC (32), it was difficult to shorten the follow-up interval of every patient because of the accompanying reduced adherence. Hence, we considered it was more efficient to implement a more intensive pattern of CEA detection on a specific group of patients with high risk. The results of this study suggest that elevated post-CEA was more likely to occur in patients with advanced age, high PLR, and elevated level of pre-CEA and preoperative CA19-9. These patients were therefore the potential beneficiaries of more frequent follow-up of serum tumor markers. Once post-CEA elevation was observed during the follow-up, detailed examinations including CT or colonoscopy were required to identify recurrence, and the patient was supposed to be re-assessed in risk stratifications to optimize subsequent therapeutic options.

Inflammation was an obvious feature of CRC and was reported to associate with serum CEA level and CRC prognosis (33, 34). Analogously, LMR, which was an indicator for systemic inflammation, was prognostic for OS and DFS, and PLR was identified as an independent predictor for post-CEA elevation in this study. However, patients with acute or chronic infectious disease, which may influence the value of inflammation-related biomarkers, were not recognized and excluded in our study. Besides, smoking status was not recorded, while it was acknowledged that false-positive CEA was highly probable in smokers (35). In general, selecting bias existed due to the single-center and retrospective nature of this study. Additionally, the follow-up duration was relatively short (median: 38 months), and this might weaken the credibility of the prognostic nomograms in predicting 5-year OS and DFS. Therefore, future studies with multicenter data, more detailed information, and longer follow-up periods were warranted to confirm our findings.

In conclusion, the post-CEA level was a strong prognostic indicator in patients with non-metastatic CRC. Our prognostic nomograms based on post-CEA and other clinical features could provide valuable information regarding the management of CRC patients. And the prediction model for post-CEA elevation could give individualized suggestions on the frequency of postoperative surveillance on serum tumor markers.

## Data Availability Statement

The raw data supporting the conclusions of this article will be made available by the authors, without undue reservation.

## Ethics Statement

The studies involving human participants were reviewed and approved by the Ethics Committee of Shanghai Jiao Tong University Affiliated Sixth People’s Hospital (No. 2018-KY-031K). The patients/participants provided their written informed consent to participate in this study.

## Author Contributions

SZ and ZW designed the study. QH, JG, and JR collected the data. SZ and JG analyzed the data. QH and NS wrote the manuscript. SZ and ZW edited the manuscript. JG and ZW supervised the project. All authors contributed to the article and approved the submitted version.

## Funding

This work was supported by Shanghai Municipal Education Commission-Gaofeng Clinical Medicine Grant Support (Grant No. 20172023).

## Conflict of Interest

The authors declare that the research was conducted in the absence of any commercial or financial relationships that could be construed as a potential conflict of interest.

## Publisher’s Note

All claims expressed in this article are solely those of the authors and do not necessarily represent those of their affiliated organizations, or those of the publisher, the editors and the reviewers. Any product that may be evaluated in this article, or claim that may be made by its manufacturer, is not guaranteed or endorsed by the publisher.
