# Supplementary material for: Clinical Significance of and Predictive Risk Factors for the Postoperative Elevation of Carcinoembryonic Antigen in Patients With Non-Metastatic Colorectal Cancer
Source: Front Oncol. 2021 Oct 7;11:741309. doi: 10.3389/fonc.2021.741309 (PMC8529031; doi:10.3389/fonc.2021.741309)
Supplement: Supplementary file 1 [file DataSheet_1.docx]

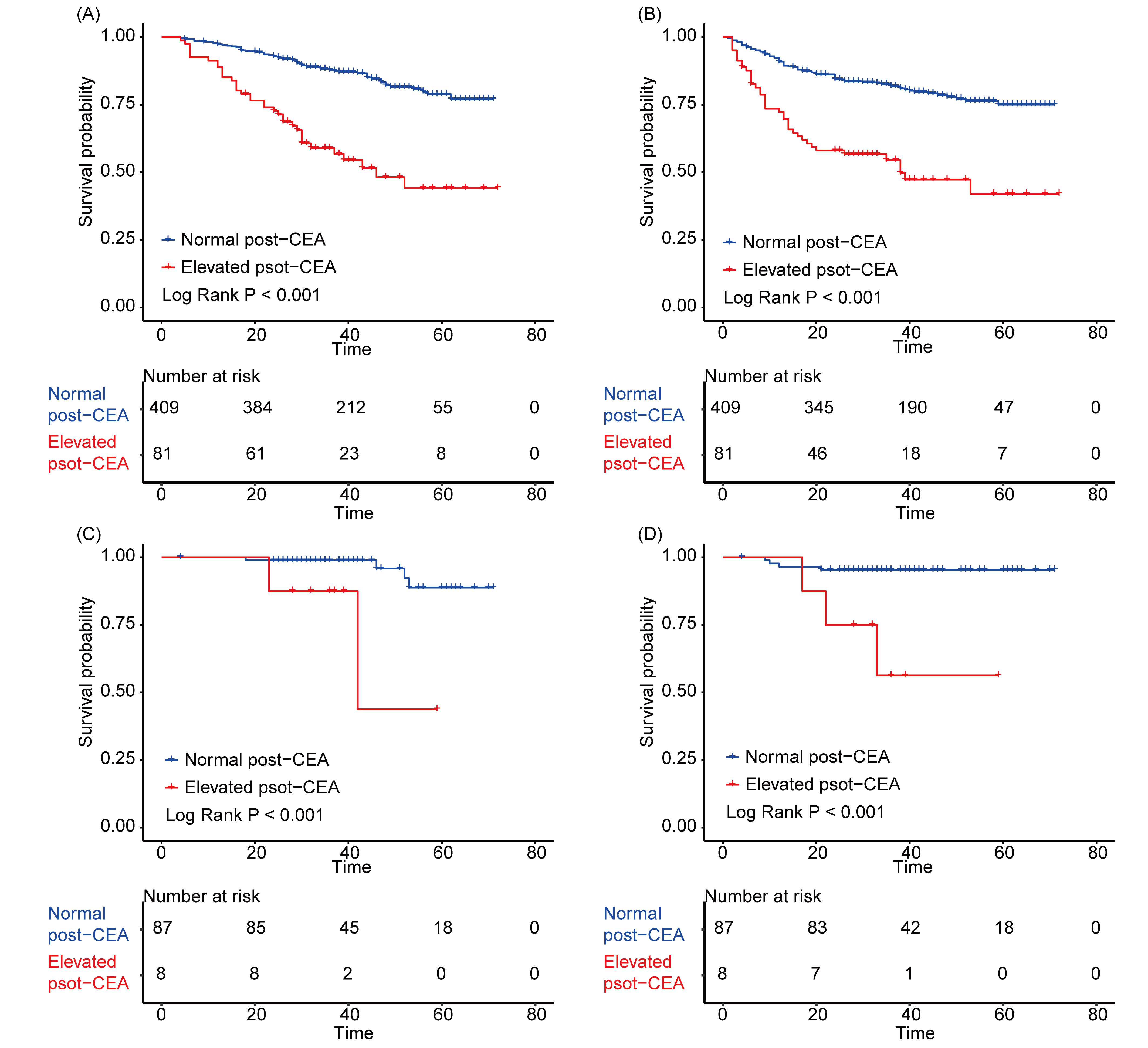


**Figure** Kaplan-Meier analysis stratified by post-CEA in patients who will be receiving adjuvant chemotherapy according to the guidelines for OS (A) and DFS (B), and in patients who will not receive adjuvant chemotherapy according to the guidelines for OS (C) and DFS (D).

**Note:** This file is only for review, not for publication.
