# Supplementary material for: Clinical Significance of and Predictive Risk Factors for the Postoperative Elevation of Carcinoembryonic Antigen in Patients With Non-Metastatic Colorectal Cancer
Source: Front Oncol. 2021 Oct 7;11:741309. doi: 10.3389/fonc.2021.741309 (PMC8529031; doi:10.3389/fonc.2021.741309)
Supplement: Supplementary file 2 [file Table_1.docx]

**Table S1.** Baseline characteristics of CRC patients in the discovery and testing cohort

| Characteristics | Dicvoery cohort  (n=352) | Testing cohort (n=233) | P value |
| --- | --- | --- | --- |
| Gender , No. (%) |  |  | 0.236 |
| Female | 140 (40%) | 105 (45%) |  |
| Male | 212 (60%) | 128 (55%) |  |
| Age , No. (%) |  |  | 0.999 |
| <60 | 99 (28%) | 65 (28%) |  |
| ≥60 | 253 (72%) | 168 (72%) |  |
| BMI , No. (%) |  |  | 0.506 |
| Underweight | 22 (6%) | 10 (4%) |  |
| Normal | 240 (68%) | 167 (72%) |  |
| Overweight | 90 (26%) | 56 (24%) |  |
| Bowel obstruction , No. (%) |  |  | 0.865 |
| No | 316 (90%) | 211 (91%) |  |
| Yes | 36 (10%) | 22 (9%) |  |
| Operation mode , No. (%) |  |  | 0.432 |
| Open | 231 (66%) | 161 (69%) |  |
| Laparoscopic | 121 (34%) | 72 (31%) |  |
| Harvested LNs , No. (%) |  |  | 0.337 |
| <12 | 111 (32%) | 64 (27%) |  |
| ≥12 | 241 (68%) | 169 (73%) |  |
| Tumor location , No. (%) |  |  | 0.463 |
| Left colon | 128 (36%) | 91 (39%) |  |
| Right colon | 90 (26%) | 65 (28%) |  |
| Rectum | 134 (38%) | 77 (33%) |  |
| Size , No. (%) |  |  | 0.873 |
| <5 cm | 200 (57%) | 130 (56%) |  |
| ≥5 cm | 152 (43%) | 103 (44%) |  |
| Histological type , No. (%) |  |  | 0.600 |
| Adenocarcinoma | 330 (94%) | 215 (92%) |  |
| Others | 22 (6%) | 18 (8%) |  |
| Differentiation , No. (%) |  |  | 0.943 |
| Well/Moderate | 256 (73%) | 168 (72%) |  |
| Poor/Undifferentiated | 96 (27%) | 65 (28%) |  |
| Lymphovascular invasion , No. (%) |  |  | 0.972 |
| Negative | 178 (51%) | 119 (51%) |  |
| Positive | 174 (49%) | 114 (49%) |  |
| Perineural invasion , No. (%) |  |  | 0.810 |
| Negative | 58 (16%) | 41 (18%) |  |
| Positive | 294 (84%) | 192 (82%) |  |
| pT stage , No. (%) |  |  | 0.593 |
| T1,T2,T3 | 198 (56%) | 125 (54%) |  |
| T4 | 154 (44%) | 108 (46%) |  |
| pN stage , No. (%) |  |  | 0.575 |
| N0 | 189 (54%) | 127 (55%) |  |
| N1 | 110 (31%) | 65 (28%) |  |
| N2 | 53 (15%) | 41 (18%) |  |
| pTNM stage , No. (%) |  |  | 0.977 |
| Stage I | 53 (15%) | 35 (15%) |  |
| Stage II | 136 (39%) | 92 (39%) |  |
| Stage III | 163 (46%) | 106 (45%) |  |
| Microsatellite status , No. (%) |  |  | 0.498 |
| pMMR | 320 (91%) | 207 (89%) |  |
| dMMR | 32 (9%) | 26 (11%) |  |
| KRAS status , No. (%) |  |  | 0.136 |
| Wild type | 107 (30%) | 89 (38%) |  |
| Mutated | 114 (32%) | 64 (27%) |  |
| Unknown | 131 (37%) | 80 (34%) |  |
| NLR , No. (%) |  |  | 0.825 |
| <3.08 | 240 (68%) | 156 (67%) |  |
| ≥3.08 | 112 (32%) | 77 (33%) |  |
| PLR , No. (%) |  |  | 0.916 |
| <192.5 | 239 (68%) | 160 (69%) |  |
| ≥192.5 | 113 (32%) | 73 (31%) |  |
| LMR , No. (%) |  |  | 0.359 |
| <2.29 | 55 (16%) | 44 (19%) |  |
| ≥2.29 | 297 (84%) | 189 (81%) |  |
| CA125 , No. (%) |  |  | 0.089 |
| <35 | 341 (97%) | 218 (94%) |  |
| ≥35 | 11 (3%) | 15 (6%) |  |
| CA199 , No. (%) |  |  | 0.982 |
| <27 | 278 (79%) | 183 (79%) |  |
| ≥27 | 74 (21%) | 50 (21%) |  |
| pre-CEA , No. (%) |  |  | 0.545 |
| <5 | 223 (63%) | 141 (61%) |  |
| ≥5 | 129 (37%) | 92 (39%) |  |
| post-CEA , No. (%) |  |  | 0.999 |
| <5 | 298 (85%) | 198 (85%) |  |
| ≥5 | 54 (1%5) | 35 (15%) |  |
| Recurrence , No. (%) |  |  | 0.186 |
| No | 268 (76%) | 189 (81%) |  |
| Yes | 84 (24%) | 44 (19%) |  |
| Survival , No. (%) |  |  | 0.277 |
| Alive | 284 (81%) | 197 (85%) |  |
| Dead | 68 (19%) | 36 (15%) |  |

**Abbreviations:** CRC, colorectal cancer; BMI, body mass index; dMMR, deficiency in DNA mismatch repair; pMMR, proficiency in DNA mismatch repair; NLR, neutrophil to lymphocyte ratio; PLR,

platelet to lymphocyte ratio; LMR, lymphocyte to monocyte ratio; pre-CEA, preoperative carcinoembryonic antigen; post-CEA, postoperative carcinoembryonic antigen
