# Supplementary material for: Clinical Significance of and Predictive Risk Factors for the Postoperative Elevation of Carcinoembryonic Antigen in Patients With Non-Metastatic Colorectal Cancer
Source: Front Oncol. 2021 Oct 7;11:741309. doi: 10.3389/fonc.2021.741309 (PMC8529031; doi:10.3389/fonc.2021.741309)
Supplement: Supplementary file 3 [file Table_2.docx]

**Table S2.** The correlation between post-CEA levels and clinicopathological features in CRC patients

| Characteristics | Elevated  post-CEA (n=89) | Normal  post-CEA (n=456) | P value |
| --- | --- | --- | --- |
| Gender , No. (%) |  |  | 0.114 |
| Female | 30 (34%) | 215 (43%) |  |
| Male | 59 (66%) | 281 (57%) |  |
| Age , No. (%) |  |  | <0.001 |
| <60 | 10 (11%) | 154 (31%) |  |
| ≥60 | 79 (89%) | 342 (69%) |  |
| BMI , No. (%) |  |  | 0.095 |
| Underweight | 9 (10%) | 23 (5%) |  |
| Normal | 61 (69%) | 346 (70%) |  |
| Overweight | 19 (21%) | 127 (26%) |  |
| Bowel obstruction , No. (%) |  |  | 0.072 |
| No | 75 (84%) | 452 (91%) |  |
| Yes | 14 (16%) | 44 (9%) |  |
| Operation mode , No. (%) |  |  | 0.151 |
| Open | 66 (74%) | 326 (66%) |  |
| Laparoscopic | 23 (26%) | 170 (34%) |  |
| Harvested LNs , No. (%) |  |  | 0.826 |
| <12 | 28 (31%) | 147 (30%) |  |
| ≥12 | 61 (69%) | 349 (70%) |  |
| Tumor location , No. (%) |  |  | 0.024 |
| Left colon | 27 (30%) | 192 (39%) |  |
| Right colon | 34 (38%) | 121 (24%) |  |
| Rectum | 28 (31%) | 183 (37%) |  |
| Size , No. (%) |  |  | 0.692 |
| <5 cm | 48 (54%) | 282 (57%) |  |
| ≥5 cm | 41 (46%) | 214 (43%) |  |
| Histological type , No. (%) |  |  | 0.519 |
| Adenocarcinoma | 81 (91%) | 464 (94%) |  |
| Others | 8 (9%) | 32 (6%) |  |
| Differentiation , No. (%) |  |  | 0.798 |
| Well/Moderate | 66 (74%) | 358 (72%) |  |
| Poor/Undifferentiated | 23 (26%) | 138 (28%) |  |
| Lymphovascular invasion , No. (%) |  |  | 0.046 |
| Negative | 36 (40%) | 261 (53%) |  |
| Positive | 53 (60%) | 235 (47%) |  |
| Perineural invasion , No. (%) |  |  | 0.632 |
| Negative | 13 (15%) | 86 (17%) |  |
| Positive | 76 (85%) | 410 (83%) |  |
| pT stage , No. (%) |  |  | 0.026 |
| T1,T2,T3 | 39 (44%) | 284 (57%) |  |
| T4 | 50 (56%) | 212 (43%) |  |
| pN stage , No. (%) |  |  | 0.023 |
| N0 | 37 (42%) | 279 (56%) |  |
| N1 | 31 (35%) | 144 (29%) |  |
| N2 | 21 (24%) | 73 (15%) |  |
| pTNM stage , No. (%) |  |  | 0.019 |
| Stage I | 7 (8%) | 81 (16%) |  |
| Stage II | 30 (34%) | 198 (40%) |  |
| Stage III | 52 (58%) | 217 (44%) |  |
| Microsatellite status , No. (%) |  |  | 0.519 |
| pMMR | 78 (88%) | 449 (91%) |  |
| dMMR | 11 (12%) | 47 (9%) |  |
| KRAS status , No. (%) |  |  | 0.666 |
| Wild type | 33 (37%) | 163 (33%) |  |
| Mutated | 24 (27%) | 154 (31%) |  |
| Unknown | 32 (36%) | 179 (36%) |  |
| NLR , No. (%) |  |  | 0.016 |
| <3.08 | 50 (56%) | 346 (70%) |  |
| ≥3.08 | 39 (44%) | 150 (30%) |  |
| PLR , No. (%) |  |  | <0.001 |
| <192.5 | 46 (52%) | 353 (71%) |  |
| ≥192.5 | 43 (48%) | 143 (29%) |  |
| LMR , No. (%) |  |  | 0.291 |
| <2.29 | 19 (21%) | 80 (16%) |  |
| ≥2.29 | 70 (79%) | 416 (84%) |  |
| pre-CEA , No. (%) |  |  | <0.001 |
| <5 | 24 (27%) | 340 (69%) |  |
| ≥5 | 65 (73%) | 156 (31%) |  |
| CA125 , No. (%) |  |  | 0.155 |
| <35 | 82 (92%) | 477 (96%) |  |
| ≥35 | 7 (8%) | 19 (4%) |  |
| CA199 , No. (%) |  |  | <0.001 |
| <27 | 53 (60%) | 408 (82%) |  |
| ≥27 | 36 (40%) | 88 (18%) |  |
| Recurrence , No. (%) |  |  | <0.001 |
| No | 47 (53%) | 410 (83%) |  |
| Yes | 42 (47%) | 86 (17%) |  |
| Survival , No. (%) |  |  | <0.001 |
| Alive | 51 (57%) | 430 (87%) |  |
| Dead | 38 (43%) | 66 (13%) |  |

**Abbreviations:** CRC, colorectal cancer; BMI, body mass index; dMMR, deficiency in DNA mismatch repair; pMMR, proficiency in DNA mismatch repair; NLR, neutrophil to lymphocyte ratio; PLR, platelet to lymphocyte ratio; LMR, lymphocyte to monocyte ratio; pre-CEA, preoperative carcinoembryonic antigen; post-CEA, postoperative carcinoembryonic antigen
