# Supplementary material for: Clinical Significance of and Predictive Risk Factors for the Postoperative Elevation of Carcinoembryonic Antigen in Patients With Non-Metastatic Colorectal Cancer
Source: Front Oncol. 2021 Oct 7;11:741309. doi: 10.3389/fonc.2021.741309 (PMC8529031; doi:10.3389/fonc.2021.741309)
Supplement: Supplementary file 4 [file Table_3.docx]

**Table S3.** Univariate and multivariate analysis of variables associated with OS of CRC patients in the discovery cohort

| Variables | Univariate analysis | | Multivariate analysis | |
| --- | --- | --- | --- | --- |
|  | HR (95% CI) | P value | HR (95% CI) | P value |
| Gender |  |  |  |  |
| Female | Reference | - |  |  |
| Male | 1.12 (0.684-1.834) | 0.652 |  |  |
| Age |  |  |  |  |
| <60 | Reference | - |  |  |
| ≥60 | 1.505 (0.858-2.64) | 0.154 |  |  |
| BMI |  |  |  |  |
| Underweight | Reference | - | Reference | - |
| Normal | 0.313 (0.157-0.625) | 0.001 | 0.308 (0.148-0.642) | 0.002 |
| Overweight | 0.292 (0.131-0.652) | 0.003 | 0.368 (0.157-0.862) | 0.021 |
| Bowel obstruction |  |  |  |  |
| No | Reference | - |  |  |
| Yes | 1.826 (0.957-3.486) | 0.068 |  |  |
| Operation mode |  |  |  |  |
| Open | Reference | - | Reference | - |
| Laparoscopic | 0.468 (0.260-0.843) | 0.011 | 0.617 (0.328-1.16) | 0.134 |
| Harvested LNs |  |  |  |  |
| <12 | Reference | - |  |  |
| ≥12 | 1.012 (0.605-1.693) | 0.965 |  |  |
| Tumor location |  |  |  |  |
| Left colon | Reference | - |  |  |
| Right colon | 1.049 (0.560-1.963) | 0.882 |  |  |
| Rectum | 1.191 (0.686-2.068) | 0.535 |  |  |
| Size |  |  |  |  |
| <5 cm | Reference | - |  |  |
| ≥5 cm | 1.077 (0.668-1.736) | 0.761 |  |  |
| Histological type |  |  |  |  |
| Adenocarcinoma | Reference | - |  |  |
| Others | 1.076 (0.432-2.681) | 0.874 |  |  |
| Differentiation |  |  |  |  |
| Well/Moderate | Reference | - | Reference | - |
| Poor/Undifferentiated | 1.906 (1.168-3.112) | 0.010 | 1.774 (1.056-2.98) | 0.030 |
| Lymphovascular invasion |  |  |  |  |
| Negative | Reference | - | Reference | - |
| Positive | 1.988 (1.213-3.256) | 0.006 | 0.791 (0.392-1.599) | 0.514 |
| Perineural invasion |  |  |  |  |
| Negative | Reference | - |  |  |
| Positive | 1.332 (0.661-2.687) | 0.423 |  |  |
| pT stage |  |  |  |  |
| T1,T2,T3 | Reference | - |  |  |
| T4 | 2.133 (1.295-3.513) | 0.003 | 1.366 (0.793-2.353) | 0.261 |
| pN stage |  |  |  |  |
| N0 | Reference | - | Reference | - |
| N1 | 2.539 (1.458-4.422) | 0.001 | 2.873 (1.413-5.841) | 0.004 |
| N2 | 3.194 (1.695-6.020) | 0.000 | 4.633 (1.873-11.457) | 0.001 |
| Microsatellite status |  |  |  |  |
| pMMR | Reference | - |  |  |
| dMMR | 0.652 (0.237-1.791) | 0.407 |  |  |
| KRAS status |  |  |  |  |
| Wild type | Reference | - |  |  |
| Mutated | 1.017 (0.546-1.891) | 0.959 |  |  |
| Unknown | 0.906 (0.499-1.644) | 0.745 |  |  |
| NLR |  |  |  |  |
| <3.08 | Reference | - | Reference | - |
| ≥3.08 | 1.616 (0.999-2.613) | 0.050 | 0.839 (0.42-1.676) | 0.619 |
| PLR |  |  |  |  |
| <192.5 | Reference | - | Reference | - |
| ≥192.5 | 2.053 (1.275-3.307) | 0.003 | 1.191 (0.652-2.175) | 0.569 |
| LMR |  |  |  |  |
| <2.29 | Reference | - |  |  |
| ≥2.29 | 0.373 (0.221-0.629) | <0.001 | 0.358 (0.175-0.734) | 0.005 |
| CA125 |  |  |  |  |
| <35 | Reference | - |  |  |
| ≥35 | 1.609 (0.505-5.121) | 0.421 |  |  |
| CA199 |  |  |  |  |
| <27 | Reference | - | Reference | - |
| ≥27 | 2.315 (1.399-3.829) | 0.001 | 1.754 (0.997-3.084) | 0.051 |
| pre-CEA |  |  |  |  |
| <5 | Reference | - | Reference | - |
| ≥5 | 1.633 (1.013-2.633) | 0.044 | 0.863 (0.493-1.509) | 0.605 |
| post-CEA |  |  |  |  |
| <5 | Reference | - | Reference | - |
| ≥5 | 4.591 (2.807-7.507) | <0.001 | 3.614 (2.045-6.388) | <0.001 |

**Abbreviations:** CRC, colorectal cancer; OS, overall survival; HR, hazard ratio; CI, confidence interval; BMI, body mass index; dMMR, deficiency in DNA mismatch repair; pMMR, proficiency in DNA mismatch repair; NLR, neutrophil to lymphocyte ratio; PLR, platelet to lymphocyte ratio; LMR, lymphocyte to monocyte ratio; pre-CEA, preoperative carcinoembryonic antigen; post-CEA, postoperative carcinoembryonic antigen
