# Supplementary material for: Clinical Significance of and Predictive Risk Factors for the Postoperative Elevation of Carcinoembryonic Antigen in Patients With Non-Metastatic Colorectal Cancer
Source: Front Oncol. 2021 Oct 7;11:741309. doi: 10.3389/fonc.2021.741309 (PMC8529031; doi:10.3389/fonc.2021.741309)
Supplement: Supplementary file 5 [file Table_4.docx]

**Table S4.** Univariate and multivariate analysis of variables associated with DFS of CRC patients in the discovery cohort

| Variables | Univariate analysis | | Multivariate analysis | |
| --- | --- | --- | --- | --- |
|  | HR (95% CI) | P value | HR (95% CI) | P value |
| Gender |  |  |  |  |
| Female | Reference | - |  |  |
| Male | 1.262 (0.805-1.979) | 0.311 |  |  |
| Age |  |  |  |  |
| <60 | Reference | - |  |  |
| ≥60 | 1.29 (0.787-2.117) | 0.313 |  |  |
| BMI |  |  |  |  |
| Underweight | Reference | - | Reference | - |
| Normal | 0.581 (0.277-1.217) | 0.150 | 0.448 (0.207-0.969) | 0.041 |
| Overweight | 0.401 (0.172-0.939) | 0.035 | 0.393 (0.162-0.955) | 0.039 |
| Bowel obstruction |  |  |  |  |
| No | Reference | - |  |  |
| Yes | 1.586 (0.861-2.924) | 0.139 |  |  |
| Operation mode |  |  |  |  |
| Open | Reference | - |  |  |
| Laparoscopic | 0.812 (0.511-1.29) | 0.377 |  |  |
| Harvested LNs |  |  |  |  |
| <12 | Reference | - |  |  |
| ≥12 | 0.830 (0.531-1.297) | 0.414 |  |  |
| Tumor location |  |  |  |  |
| Left colon | Reference | - |  |  |
| Right colon | 0.82 (0.457-1.47) | 0.504 |  |  |
| Rectum | 1.131 (0.697-1.837) | 0.618 |  |  |
| Size |  |  |  |  |
| <5 cm | Reference | - |  |  |
| ≥5 cm | 1.036 (0.673-1.593) | 0.873 |  |  |
| Histological type |  |  |  |  |
| Adenocarcinoma | Reference | - |  |  |
| Others | 0.888 (0.359-2.196) | 0.797 |  |  |
| Differentiation |  |  |  |  |
| Well/Moderate | Reference | - | Reference | - |
| Poor/Undifferentiated | 1.960 (1.264-3.038) | 0.003 | 1.667 (1.051-2.643) | 0.030 |
| Lymphovascular invasion |  |  |  |  |
| Negative | Reference | - | Reference | - |
| Positive | 2.074 (1.327-3.241) | 0.001 | 0.736 (0.387-1.401) | 0.351 |
| Perineural invasion |  |  |  |  |
| Negative | Reference | - |  |  |
| Positive | 1.749 (0.876-3.491) | 0.113 |  |  |
| pT stage |  |  |  |  |
| T1,T2,T3 | Reference | - | Reference | - |
| T4 | 2.224 (1.427-3.468) | <0.001 | 1.545 (0.963-2.479) | 0.071 |
| pN stage |  |  |  |  |
| N0 | Reference | - | Reference | - |
| N1 | 2.671 (1.593-4.48) | <0.001 | 2.792 (1.458-5.349) | 0.002 |
| N2 | 4.368 (2.508-7.608) | <0.001 | 6.133 (2.781-13.526) | <0.001 |
| Microsatellite status |  |  |  |  |
| pMMR | Reference | - |  |  |
| dMMR | 0.497 (0.182-1.358) | 0.173 |  |  |
| KRAS status |  |  |  |  |
| Wild type | Reference | - |  |  |
| Mutated | 1.256 (0.73-2.163) | 0.410 |  |  |
| Unknown | 0.959 (0.556-1.656) | 0.882 |  |  |
| NLR |  |  |  |  |
| <3.08 | Reference | - | Reference | - |
| ≥3.08 | 1.771 (1.151-2.725) | 0.009 | 0.967 (0.53-1.765) | 0.914 |
| PLR |  |  |  |  |
| <192.5 | Reference | - | Reference | - |
| ≥192.5 | 2.242 (1.461-3.44) | <0.001 | 1.353 (0.79-2.319) | 0.271 |
| LMR |  |  |  |  |
| <2.29 | Reference | - | Reference | - |
| ≥2.29 | 0.383 (0.238-0.616) | <0.001 | 0.392 (0.205-0.748) | 0.005 |
| CA125 |  |  |  |  |
| <35 | Reference | - |  |  |
| ≥35 | 1.271 (0.402-4.026) | 0.683 |  |  |
| CA199 |  |  |  |  |
| <27 | Reference | - | Reference | - |
| ≥27 | 1.629 (1.008-2.633) | 0.046 | 1.103 (0.662-1.837) | 0.706 |
| pre-CEA |  |  |  |  |
| <5 | Reference | - |  |  |
| ≥5 | 1.121 (0.722-1.742) | 0.611 |  |  |
| post-CEA |  |  |  |  |
| <5 | Reference | - | Reference | - |
| ≥5 | 3.54 (2.235-5.606) | <0.001 | 3.072 (1.861-5.073) | <0.001 |

**Abbreviations:** CRC, colorectal cancer; DFS, disease-free survival; HR, hazard ratio; CI, confidence interval; BMI, body mass index; dMMR, deficiency in DNA mismatch repair; pMMR, proficiency in DNA mismatch repair; NLR, neutrophil to lymphocyte ratio; PLR, platelet to lymphocyte ratio; LMR, lymphocyte to monocyte ratio; pre-CEA, preoperative carcinoembryonic antigen; post-CEA, postoperative carcinoembryonic antigen
