# Supplementary material for: Clinical Significance of and Predictive Risk Factors for the Postoperative Elevation of Carcinoembryonic Antigen in Patients With Non-Metastatic Colorectal Cancer
Source: Front Oncol. 2021 Oct 7;11:741309. doi: 10.3389/fonc.2021.741309 (PMC8529031; doi:10.3389/fonc.2021.741309)
Supplement: Supplementary file 6 [file Table_5.docx]

**Table S5.** Univariate and multivariate analysis of variables associated with post-CEA elevation in the discovery cohort

| Variables | Univariate analysis | | Multivariate analysis | |
| --- | --- | --- | --- | --- |
|  | OR (95% CI) | P value | OR (95% CI) | P value |
| Gender |  |  |  |  |
| Female | Reference | - |  |  |
| Male | 1.693 (0.918-3.248) | 0.100 |  |  |
| Age |  |  |  |  |
| <60 | Reference | - | Reference | - |
| ≥60 | 4.516 (1.907-13.318) | 0.002 | 4.675 (1.844-14.531) | 0.003 |
| BMI |  |  |  |  |
| Underweight | Reference | - |  |  |
| Normal | 0.660 (0.244-2.100) | 0.439 |  |  |
| Overweight | 0.425 (0.132-1.506) | 0.160 |  |  |
| Bowel obstruction |  |  |  |  |
| No | Reference | - |  |  |
| Yes | 2.007 (0.844-4.41) | 0.095 |  |  |
| Operation mode |  |  |  |  |
| Open | Reference | - | Reference | - |
| Laparoscopic | 0.495 (0.24-0.954) | 0.044 | 0.545 (0.237-1.190) | 0.137 |
| Harvested LNs |  |  |  |  |
| <12 | Reference | - |  |  |
| ≥12 | 0.907 (0.495-1.711) | 0.757 |  |  |
| Tumor location |  |  |  |  |
| Left colon | Reference | - | Reference | - |
| Right colon | 2.152 (1.039-4.545) | 0.040 | 1.323 (0.573-3.079) | 0.511 |
| Rectum | 1.245 (0.604-2.604) | 0.554 | 1.550 (0.683-3.595) | 0.298 |
| Size |  |  |  |  |
| <5 cm | Reference | - |  |  |
| ≥5 cm | 0.972 (0.536-1.741) | 0.924 |  |  |
| Histological type |  |  |  |  |
| Adenocarcinoma | Reference | - |  |  |
| Others | 0.864 (0.198-2.653) | 0.819 |  |  |
| Differentiation |  |  |  |  |
| Well/Moderate | Reference | - |  |  |
| Poor/Undifferentiated | 0.922 (0.463-1.747) | 0.809 |  |  |
| Lymphovascular invasion |  |  |  |  |
| Negative | Reference | - | Reference | - |
| Positive | 1.919 (1.065-3.537) | 0.033 | 1.762 (0.912-3.480) | 0.096 |
| Perineural invasion |  |  |  |  |
| Negative | Reference | - |  |  |
| Positive | 1.159 (0.54-2.785) | 0.721 |  |  |
| pT stage |  |  |  |  |
| T1,T2,T3 | Reference | - |  |  |
| T4 | 1.470 (0.821-2.64) | 0.194 |  |  |
| pN stage |  |  |  |  |
| N0 | Reference | - |  |  |
| N1 | 1.791 (0.93-3.444) | 0.079 |  |  |
| N2 | 1.988 (0.869-4.352) | 0.092 |  |  |
| Microsatellite status |  |  |  |  |
| pMMR | Reference | - |  |  |
| dMMR | 1.308 (0.467-3.156) | 0.576 |  |  |
| KRAS status |  |  |  |  |
| Wild type | Reference | - |  |  |
| Mutated | 0.636 (0.290-1.364) | 0.249 |  |  |
| Unknown | 1.053 (0.536-2.095) | 0.881 |  |  |
| NLR |  |  |  |  |
| <3.08 | Reference | - | Reference | - |
| ≥3.08 | 1.909 (1.050-3.446) | 0.032 | 1.031 (0.471-2.223) | 0.939 |
| PLR |  |  |  |  |
| <192.5 | Reference | - |  |  |
| ≥192.5 | 2.465 (1.365-4.458) | 0.003 |  |  |
| LMR |  |  |  |  |
| <2.29 | Reference | - | Reference | - |
| ≥2.29 | 0.59 (0.294-1.252) | 0.150 | 2.168 (1.016-4.657) | 0.045 |
| CA125 |  |  |  |  |
| <35 | Reference | - |  |  |
| ≥35 | 1.235 (0.185-4.963) | 0.791 |  |  |
| CA199 |  |  |  |  |
| <27 | Reference | - | Reference | - |
| ≥27 | 3.593 (1.926-6.661) | 0.000 | 2.294 (1.138-4.583) | 0.019 |
| pre-CEA |  |  |  |  |
| <5 | Reference | - | Reference | - |
| ≥5 | 5.402 (2.915-10.433) | <0.001 | 4.097 (2.082-8.328) | <0.001 |

**Abbreviations:** post-CEA, postoperative carcinoembryonic antigen; OR, odds ratio; CI,confidence interval; BMI, body mass index; dMMR, deficiency in DNA mismatch repair; pMMR, proficiency in DNA mismatch repair; NLR, neutrophil to lymphocyte ratio; PLR, platelet to lymphocyte ratio; LMR, lymphocyte to monocyte ratio; pre-CEA, preoperative carcinoembryonic antigen;
